# Supplementary material for: Neonatal birth trauma and associated factors in low and middle-income countries: A systematic review and meta-analysis
Source: PLoS One. 2024 Mar 21;19(3):e0298519. doi: 10.1371/journal.pone.0298519 (PMC10957092; doi:10.1371/journal.pone.0298519)
Supplement: S1 Table — JBI’s critical appraisal tools: (A) Descriptive cross-sectional studies. (B) Analytical cross-sectional studies. (C) case-control studies. (DOCX) [file pone.0298519.s002.docx]

**S1 Table A** : Quality assessment of studies using JBI’s critical appraisal tools designed for Descriptive cross-sectional study

| Study | JBI’s critical appraisal questions | | | | | | | | | | | | | | | | Score | Overall Appraisal |
| --- | --- | --- | --- | --- | --- | --- | --- | --- | --- | --- | --- | --- | --- | --- | --- | --- | --- | --- |
|  | Q1 | Q2 | Q3 | | Q4 | | Q5 | | Q6 | | Q7 | | Q8 | | Q9 | |  |  |
| Mah et al | Y | Y | | Y | | Y | | U | | Y | | Y | | Y | | Y | 8 | Included |
| Abdul-mumin et al | Y | Y | | Y | | Y | | Y | | Y | | Y | | U | | Y | 8 | Included |
| Pius et al | Y | Y | | Y | | Y | | Y | | Y | | Y | | Y | | Y | 8 | Included |
| West et al | Y | Y | | Y | | Y | | Y | | Y | | Y | | N | | Y | 8 | Included |
| Emeka et al | Y | Y | | Y | | Y | | Y | | Y | | Y | | Y | | Y | 9 | Included |
| Warke et al | Y | Y | | Y | | Y | | Y | | Y | | Y | | Y | | Y | 9 | Included |
| Zama et *al* | Y | Y | | Y | | N | | Y | | Y | | Y | | Y | | Y | 8 | Included |
| Mosavat et *al* | Y | Y | | Y | | Y | | y | | N | | Y | | Y | | Y | 9 | Included |
| Shanthi et al | Y | Y | | Y | | Y | | Y | | N | | Y | | N | | Y | 8 | Included |
| Awari et al | Y | Y | | Y | | Y | | Y | | Y | | Y | | Y | | N | 8 | Included |
| Rezaie et al | Y | Y | | Y | | Y | | Y | | Y | | Y | | N | | Y | 8 | Included |
| Enyindah et al | Y | Y | | Y | | Y | | Y | | Y | | Y | | N | | Y | 8 | Included |
| Adegbehingbe et al | Y | Y | | Y | | Y | | Y | | Y | | Y | | N | | Y | 8 | Included |
| Osinaike et al | Y | Y | | Y | | Y | | Y | | Y | | Y | | N | | Y | 8 | Included |
| Fabamwo et al | Y | Y | | Y | | Y | | Y | | Y | | Y | | N | | Y | 8 | Included |
| Njokanma and Kehinde | Y | Y | | Y | | Y | | Y | | Y | | Y | | N | | Y | 8 | Included |
| Okoro and Oriji | Y | Y | | Y | | Y | | Y | | Y | | Y | | N | | Y | 8 | Included |
| Danso and Shaka | Y | Y | | Y | | Y | | Y | | Y | | Y | | N | | Y | 8 | Included |
| Uchenna et al | Y | Y | | Y | | Y | | Y | | Y | | Y | | N | | Y | 8 | Included |
| Gorashi et al | Y | Y | | Y | | Y | | Y | | Y | | Y | | N | | Y | 8 | Included |
| Esmailpour et al | Y | Y | | Y | | Y | | Y | | Y | | Y | | N | | Y | 8 | Included |
| Ray et al | Y | Y | | Y | | Y | | Y | | Y | | Y | | N | | Y | 8 | Included |
| Prabhu et al | Y | Y | | Y | | Y | | Y | | Y | | Y | | N | | Y | 8 | Included |
| Benjamin et al | Y | Y | | Y | | Y | | Y | | Y | | Y | | N | | Y | 8 | Included |
| Shabbir et al | Y | Y | | Y | | Y | | Y | | Y | | Y | | N | | Y | 8 | Included |
| Tibebeu et al | Y | Y | | Y | | Y | | Y | | Y | | Y | | N | | Y | 8 | Included |

Y –Yes;N-No;U -Unclear-Question. Overall score is calculated by counting the number of Y’s in each row.Q1=Was the sample frame appropriate to address the target population? Q2=Were study participants sampled in an appropriate way? Q3=Was the sample size adequate? Q4=Were the study subjects and the setting described in detail? Q5=Was the data analysis conducted with sufficient coverage of the identified sample? Q6=Were valid methods used for the identification of the condition? Q7=Was the condition measured in a standard, reliable way for all participants? Q8=Was there appropriate statistical analysis? Q9=Was the response rate adequate, and if not, was the low response rate managed appropriately?

**S1 Table B**: Quality assessment of studies using JBI’s critical appraisal tools designed for Analytical cross-sectional study

| Study | JBI’s critical appraisal questions | | | | | | | | Score | Overall Appraisal |
| --- | --- | --- | --- | --- | --- | --- | --- | --- | --- | --- |
|  | Q1 | Q2 | Q3 | Q4 | Q5 | Q6 | Q7 | Q8 |  |  |
| Tesfaye et al | Y | y | y | y | y | y | y | y | 8 | Included |
| Yemane et al | y | y | y | y | y | y | y | y | 8 | Included |
| Biset et al | Y | y | y | y | y | y | y | y | 8 | Included |
| Tolosa et al | Y | y | y | y | y | y | y | y | 8 | Included |
| Belay et al | y | y | y | y | y | y | y | y | 8 | Included |
| Linder et *al* | y | y | y | y | y | y | y | y | 8 | Included |
| Phuengphaeng et al | y | y | y | y | y | y | y | y | 8 | Included |
| Borna et al | y | y | y | y | y | y | y | y | 8 | Included |
| Abedzadeh et *al* | y | n | y | y | y | y | y | y | 7 | Included |

Y –Yes;N-No;U -Unclear-Question. Overall score is calculated by counting the number of Y’s in

For analytical cross-sectional study, the JBI checklist assessed the following questions

Major components :

1. Were the criteria for inclusion in the sample clearly defined?

2. Were the study subjects and the setting described in detail?

3. Was the exposure measured in a valid and reliable way?

4. Were objective, standard criteria used for measurement of the condition?

5. Were confounding factors identified?

6. Were strategies to deal with confounding factors stated?

7. Were the outcomes measured in a valid and reliable way?

8. Was appropriate statistical analysis used?

**S1 Table C: Quality assessment of studies using JBI’s critical appraisal tools designed for case-control study design**

| Study | JBI’s critical appraisal questions | | | | | | | |  |  | Score | Overall Appraisal |
| --- | --- | --- | --- | --- | --- | --- | --- | --- | --- | --- | --- | --- |
|  | Q1 | Q2 | Q3 | Q4 | Q5 | Q6 | Q7 | Q8 | Q9 | Q 10 |  |  |
| Hameed and Izzet | Y | n | y | y | y | y | y | y | y | y | 9 | Included |
| Basiri et al | y | n | y | y | y | y | y | y | y | y | 9 | Included |

Y –Yes;N-No;U -Unclear-Question. An overall score is calculated by counting the number of Y’s in

For the case-control study, the JBI checklist assessed the following questions

Major components :

Q1) Were the groups comparable other than the presence of disease in cases or the absence of disease in controls? (Q2) Were cases and controls matched appropriately? (Q3) Were the same criteria used for the identification of cases and controls? (Q4) Was exposure measured in a standard, valid, and reliable way? (Q5) Was exposure measured in the same way for cases and controls? (Q6) Were confounding factors identified? (Q7) Were strategies to deal with confounding factors stated? (Q8) Were outcomes assessed in a standard, valid, and reliable way for cases and controls? (Q9) Was the exposure period of interest long enough to be meaningful? (Q10) Was appropriate statistical analysis used?
