# Supplementary material for: Neonatal birth trauma and associated factors in low and middle-income countries: A systematic review and meta-analysis
Source: PLoS One. 2024 Mar 21;19(3):e0298519. doi: 10.1371/journal.pone.0298519 (PMC10957092; doi:10.1371/journal.pone.0298519)
Supplement: Attachment — Submitted filename: Peer Review Report.pdf [file pone.0298519.s007.pdf]

Peer Review Report

Title: Neonatal birth trauma and associated factors in low and middle-income countries: A systematic review and meta-analysis

General Comments:

The manuscript titled "Neonatal birth trauma and associated factors in low and middle-income countries: A systematic review and meta-analysis" provides valuable insights into neonatal birth trauma in low and middle-income countries. The language and organization of the manuscript are clear and concise, making it accessible to a wide audience. However, there are a few areas where additional information or clarification would enhance the manuscript's impact. With some major revisions and additions, this manuscript has the potential to make a valuable contribution to the field.

Specific Comments:

Abstract:

a. Could you provide more information on the methods used to search for articles and select studies for inclusion in the meta-analysis? This would help readers understand the rigor of the study selection process.

b. The abstract mentions the identification of potential contributing factors for birth trauma. Could you elaborate on the specific factors identified and their respective effect sizes? Providing this information would give readers a clearer understanding of the magnitude and significance of the identified factors.

Introduction and Background:

a. Can the authors provide a more comprehensive overview of the current state of neonatal birth trauma in low and middle-income countries? It would be helpful to include statistics or prevalence rates, if available. Additionally, are there any specific regions or populations within these countries that are more affected? Providing this information would give readers a better context for understanding the significance of the study.

b. It would be beneficial to include a discussion on the impact of birth trauma on neonatal morbidity and mortality in low and middle-income countries, as well as the economic burden associated with it. This discussion would help illustrate the broader implications of the research findings.

Methods:

a. Please provide more details on the search strategy used to identify relevant articles. Were any specific inclusion or exclusion criteria applied? Including this information would enhance the transparency and reproducibility of the study.

b. How was the quality of the included studies assessed? Were any measures taken to address potential bias or heterogeneity among the studies? Describing the quality assessment process and any steps taken to address bias or heterogeneity would strengthen the study's methodology.

c. Can the authors provide a rationale for selecting the Dersimonian-Laird model as the random-effects model for the meta-analysis? Justifying the choice of this model would help readers understand the statistical approach employed.

Results:

a. The authors report a high pooled cumulative incidence of birth trauma in low and middle-income countries. Can they discuss the potential reasons for this high incidence and how it compares to rates in high-income countries? Exploring the factors contributing to the high incidence and comparing it to rates in high-income countries would provide valuable insights into the unique challenges faced by low and middle-income countries.

b. It would be interesting to know if there are any temporal trends in the incidence of birth trauma in these countries. Did the authors find any studies reporting changes over time? Including information on temporal trends would enhance the understanding of the evolving situation and potential improvements over time.

c. The factors associated with neonatal birth trauma mentioned in the results are important. However, it would be beneficial to discuss the clinical implications of these factors and how they can inform preventive strategies in low and middle-income countries. Providing this discussion would help bridge the gap between research findings and practical applications.

Discussion:

a. The discussion could be strengthened by providing a more detailed analysis of the identified risk factors and their implications. Are there any modifiable factors that could be targeted to reduce the incidence of birth trauma? Including this analysis would provide actionable insights for healthcare providers and policymakers.

b. What are the potential limitations of the included studies that may affect the generalizability of the findings? Are there any gaps in the current literature that future research should address? Discussing the limitations and gaps in the literature would help readers understand the scope and applicability of the study's findings.
