# Supplementary material for: Neonatal birth trauma and associated factors in low and middle-income countries: A systematic review and meta-analysis
Source: PLoS One. 2024 Mar 21;19(3):e0298519. doi: 10.1371/journal.pone.0298519 (PMC10957092; doi:10.1371/journal.pone.0298519)
Supplement: Attachment — Submitted filename: PLOS ONE REVIEWERS COMMENT.docx [file pone.0298519.s006.docx]

**TITLE** – Appropriate.

**Abstract-** Well written with minor correction of typographical error (38 or 37 studied articles)

**Introduction**- Fairly written and justification for conducting the study was highlighted.

**Methodology-** This is quite detail and the chosen method will achieve the set-out objectives of the study. Statistical analysis was reflected in the results.

**Results-** The results was well presented but the following observations were made:

1. Page 13, paragraph 2 line 2—Insert There were 365,547 live births instead of –About 365,547 live births.
2. Page 15 line 2—data were abstracted from 35 articles ( Is it 35 or 37 articles?).
3. Page 19 line1: from 1985-2023 but Title for the figure indicates from 2008-2023. This needs to be corrected.
4. Page 20 line 1, 8, and 10: why the use of the word about in the case of an absolute number? It is more appropriate to use these words in the derived figures eg in percentages and so on.
5. Page 21 line 14 insert—assisted vaginal delivery

**DISCUSSION**: The authors did not DISCUSS the findings adequately. In most cases, it most often just restated the findings and did literature review.

**CONCLUSION/RECOMMENDATION-** Guarded recommendations were made, but however, there was also no mention of the focus future studies.

**General Comment-**

The research topic is quite topical and relevant to the practice of obstetrics and paediatrics. The authors have made commendable effort towards addressing the research objectives, but some issues particularly relating to the results and discussion need to be revised by the authors, to further improve the manuscript.
